# Neuromodulation via Focal Radiation: Radiomodulation Update

**DOI:** 10.7759/cureus.14700

**Published:** 2021-04-26

**Authors:** M. Bret Schneider, Brian Walcott, John R. Adler

**Affiliations:** 1 Chief Medical Officer, Zap Surgical Systems, Inc., San Carlos, USA; 2 Department of Psychiatry, Stanford University School of Medicine, Stanford, USA; 3 Neurosurgery, University of Chicago, Chicago, USA; 4 Neurosurgery, Northshore University HealthSystem, Evanston, USA; 5 Department of Neurosurgery, Stanford University School of Medicine, Stanford, USA

**Keywords:** radiation, neuromodulation, radiomodulation, radiosurgery, functional disorders, psychiatric disorders, behavioral disorders

## Abstract

When radiation is focally delivered to brain tissue at sub-ablative doses, neural activity may be altered. When done at a specific brain circuit node or connection, this is referred to as “radiomodulation.” Radiation-induced effects on brain tissue, basic science, and clinical research that supports the radiomodulation hypothesis are reviewed in this article. We review progress in defining the necessary parameters in terms of dose, volumes, and anatomical location. It may be possible to deliver therapeutic neuromodulation that is non-invasive, non-destructive, and durable.

## Introduction and background

Behavioral diseases and other functional disorders result in incalculable human suffering and tremendous economic losses [1]. Recent advances in basic neuroscience inform us that the function of discrete, pathological brain circuits underlie such functional disorders [2]. With this understanding, various techniques have been developed for altering pathologic circuit function, and in doing so have fostered and validated the concept of clinical neuromodulation. By manipulating the function of these circuits at specific targets in human subjects, former hypotheses have been validated and are now the rationale for clinical neuromodulation.

Radiosurgery has been proposed as a method of producing neuromodulation by Regis et al., who noted radiosurgery effects on epileptic tissue that otherwise seemed to retain basic neurological function [3,4]. Schneider et al. proposed that the radiation-induced neuromodulation principle could be applied to targeted locations within known brain circuits for the treatment of some severe and refractory psychiatric and behavioral conditions [5]. When a focal radiation dose is administered to a node or connection within a known brain circuit for a specific intended effect, this is referred to as “radiomodulation.” More work is required to establish that this research hypothesis can be effective, safe, and durable. In particular, it will be important to establish correct network targets and the pertinent volumes and doses.

## Review

Neuromodulation

One aspect of neuromodulation is the alteration of activity levels in the brain through targeted delivery of a stimulus, e.g., electrical pulses. In this way, errant neural activity may be normalized. Neuromodulation may be applied directly to the location of a neurological problem (e.g., a seizure focus in epilepsy [6]), or to a node or edge in the brain circuit, in which modulation of the target produces an intended net output from the circuit as a whole, such as the subthalamic nucleus (STN) in Parkinson’s disease [7]. Non-invasive neuromodulation methods include repetitive transcranial magnetic stimulation (rTMS) and transcranial direct current stimulation (tDCS). Both may be effective in certain clinical conditions, such as rTMS in depression, and have low side effect profiles. However, they are relatively non-focal and thus reserved for large swaths of the superficial cortex on the brain. They lack anatomical specificity, particularly at depth, even when targeted using computerized stereotactic image guidance [8]. Furthermore, rTMS, in particular, is not practical for continuous stimulation throughout the day, every day. The ability to treat clinical conditions by modulating specifically targeted brain circuits remains limited. Surgically implanted electrical stimulation of the brain, such as deep brain stimulation (DBS) and cortical stimulation, are approved for use in most of the world for the treatment of Parkinson’s disease, essential tremor dystonia, obsessive-compulsive disorder, and epilepsy. Implanted stimulation is also promising in investigational use for chronic pain, depression, and addiction [9-11]. While potent and anatomically specific, this approach requires invasive neurosurgical procedures, which are accompanied by the inherent risk of surgery within the brain, including bleeding and infection. Further, implanted stimulators are expensive and typically require periodic hardware replacement procedures throughout the patient’s life.

From radiosurgery to radiomodulation

The virtues of these sundry neuromodulation methods range from high anatomic precision and potency to less invasive, less potent, and less anatomically specific. It would seem reasonable that the optimal neuromodulation is non-invasive and both highly focal and durable in its effect. With this objective, the authors examine the utility of non-ablative, small-field ionizing radiation delivered to brain tissue. This class of procedure is termed “radiomodulation” by a small number of researchers currently exploring the field.

Stereotactic radiosurgery has been used for decades for functional neurosurgery [12,13], including thalamotomy for chronic pain [14-16], pallidotomy for movement disorders [17], and ventral anterior capsulotomy for obsessive-compulsive disorder [18,19]. However, these procedures were intended to truncate or ablate neural connections by inducing cell death [12,13,18]. By contrast, the *sine qua non* of radiomodulation is the alteration in function of living, viable neurons and glia.

Radiation-induced effects

Almost a century of research on the biological effects of ionizing radiation on tissue has revealed five factors that are critical in determining the net effect [20,21]: (1) repair of sublethal cellular damage, (2) repopulation of cells following radiation, (3) redistribution of cells within the cell cycle, (4) reoxygenation of the surviving cells, and (5) the radiosensitivity of the tissue and cells within. Accordingly, the post-radiation period involves a complex set of changes to neurons, glia, immune cell populations, and vascular and perivascular cells.

Immediate and early radiation effects occur from the first few minutes to the first few days following exposure. Irradiated tissue absorbs energy, causing changes in enzyme activity, primarily deactivation involving nicotinamide adenine dinucleotidase, adenosine triphosphatases, acid and alkaline phosphatases, cathepsin-type C esterases, glutamate dehydrogenases and synthases, and succinate dehydrogenases. Some enzymes increase in activity due to metabolic reactions. Enzymes are destroyed via free radicals produced through changes in structure by splitting off amino acids and through denaturation. There is an increase in activity of other enzymes, an accumulation of substrates of these enzymes (primarily glycogen and glycoproteins), and changes in the distribution of electrolytes. Perhaps, most famously, there is radiation damage to the karyoplasm, with damage and loss of DNA. In neurons, there is acute edema and tigrolysis within 30 minutes as ribosomes are lost [21]. In the vasculature, arteries spasm and capillaries show endothelial cell changes within minutes of exposure, with lysosomes increasing and pinocytic vesicles multiplying. Endothelial cells are desquamated, thrombi appear, and interstitial spaces surrounding the capillaries disappear. As a consequence, there is retardation of blood flow. Among glia, astrocytes manifest the first signs of irradiation, with hypertrophic cytoplasm and feet, as well as nuclei with dilated pores. Microglia show dystrophy with pyknotic nuclei and swelling of cell bodies. Unlike astrocytes, oligodendrocytes do not show observable early changes [21]. 

Radiation-induced edema is frequently evident in clinical experience. Because magnetic resonance (MR) imaging principally reflects a shift in hydrogen resonance, it is sensitive to changes in water in the white matter. As a result, asymptomatic MR hyperintensity on T2-weighted MR imaging following radiation usually represents edema. In most cases, the edema completely resolves over time and does not equate with necrosis [22]. Post-radiation white matter changes may result from an increase in extracellular fluid rather than the locally delivered dose as inflammation has been shown to assume a neuroanatomic distribution and not a radiation dose-related distribution pattern [4].

Late radiation-induced changes may be seen after months and may continue progressively for more than a year [23]. Necrosis usually occurs one to two years after radiation, but latency as short as three months and as long as 30 years has been reported [24,25]. Vascular degeneration is manifested by telangiectasis, capillary proliferation, wall thickening, perivascular edema, thrombosis, and petechial hemorrhages. Conversely, capillaries and small venules may widen. Glial atrophy, which first appears in short-term effects, may continue for years. Leukoencephalopathy is white matter injury, with histopathology showing thinning of white matter, loss of oligodendrocytes, reactive astrogliosis, and areas of necrosis [27]. Generalized atrophy of nervous tissue probably caused by glial cell depletion without clear signs of vascular injury or necrosis can occur even at low doses and after long follow-up times [23].

These features of focal radiation-induced changes, both short-term and late-term, are exploited in the clinical setting today, mainly to control cancer cell growth or to promote the destruction of vascular formations. However, there is both animal and human research that radiation may also modulate neuronal transmission in unique pathways.

Animal studies that suggest a radiomodulation effect

Key pre-clinical studies that suggest a radiomodulation effect are summarized in Table 1. They show evidence of inhibition of sodium channels, reduced synaptic transmission, hyperpolarization of neurons, necrosis at high target volumes, reduced epileptiform spiking, reduced pre-synaptic and post-synaptic responses, reduction of inhibition, and shortened action potentials.

**Table 1:** A summary of animal studies suggesting a radiomodulation effect. DNA: deoxyribonucleic acid; FDG-PET: 18 F-fluorodeoxyglucose-positron emission tomography; GABA_A_: gamma-aminobutyric acid type A; MRI: magnetic resonance imaging; PET: positron emission tomography

Reference	Paradigm	Effect observed
Mullin et al. [27]	1-100 Gy to the whole brain of a live rat. Observations ex-vivo immediately post-radiation	Radiation causes an inhibitory effect on voltage-sensitive sodium channels
Tolliver et al. [28]	Guinea pig at various dose rates. Observations ex-vivo in hippocampal slice immediately post-radiation	Reduced synaptic transmission efficiency
Pellmar et al. [29]	Guinea pig hippocampus: 5-65 Gy to the whole brain of a live animal. Observations in slice immediately post-radiation	Radiation causes chronic hyperpolarization of neurons and reduced ability of synaptic potential to generate a spike; however, synaptic efficiency increases
Yamaguchi et al. [30]	Dog: 15 Gy to the whole brain. Observations 3-30 months post-radiation	Small cell infiltration after 6 months. Necrosis at 9-15 months, with vascular narrowing. More cells in the reproductive phase post-radiation. DNA transcription at a maximum after 9 months and a minimum after 3 months
Chen et al. [31]	Rat epilepsy model: 20 Gy and 40 Gy gamma knife with 4 mm collimator	Synaptically driven firing unchanged. Epileptiform spiking decreased by 55%, with effect sustained for months. Reduced seizure frequency. Cell death in 7% of the targeted tissue
Brisman et al. [32]	Rat hippocampus: 90 Gy proton beam to rat brain, observations 3 months post-radiation	Higher voltages required to elicit post-synaptic potentials. Reduced response pre-synaptically and post-synaptically.
Dagne et al. [33]	Rat: whole-brain radiation at 60 Gy, observations at 1 day and 1 week post-radiation	60 Gy produced a reduction of GABA_A_ slow inhibition
Yeh et al. [34]	Pigs: 10-120 Gy 7.5 mm diameter targets in M1 and internal capsule. PET and MRI: baseline to 9 months post-radiation	Doses 60 Gy and above reduces FDG-PET signal at M1 target. Doses 10-40 Gy increases PET signal
Gilly et al. [35]	Squid: 140-300 Gy to 9 mm diameter target on stellate ganglion 24 hours post-radiation	Action potential recorded from the giant motor axon in response to electrical stimulation showed an increased maximum rate of fall and a shortened action potential duration

Any of these effects observed could plausibly result in up-regulation or down-regulation of the targeted brain region. For example, the inhibition of sodium channels in an inhibitory brain circuit node would plausibly result in net up-regulation of the output of that node.

**Figure 1:**
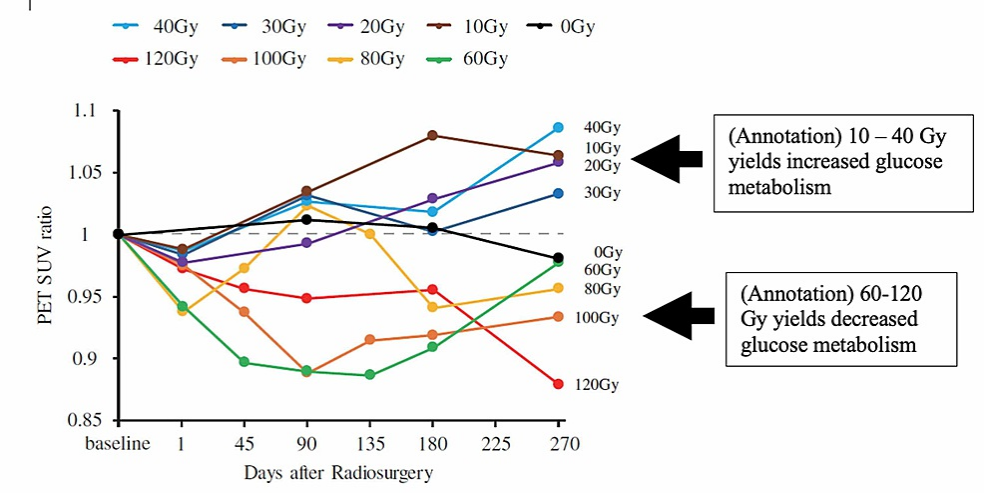
Yeh et al. showed 10-40 Gy increases and 60-120 Gy decreases in metabolism by PET in a focal cortical brain region. A 7.5-mm diameter radiosurgical target in the left primary motor cortex. FDG-PET signal at target relative to the contralateral region followed up to 270 days post-radiation. FDG-PET: 18 F-fluorodeoxyglucose positron emission tomography; M1: primary motor cortex; PET: positron emission tomography; SUV: standardized uptake value at target zone versus contralateral zone Reprinted with permission with annotations (Yeh et al. [34])

Animal studies have also suggested a novel bimodal effect of radiation on motor cortex activity, depending upon the dose. Yeh et al. utilized 18 F-fluorodeoxyglucose-positron emission tomography (FDG-PET) imaging to identify areas of increased or decrease brain metabolism following stereotatic radiosurgery in a healthy Lee Sung miniature pig model [34]. Following the identification of the left primary motor cortex (M1) nine pigs were treated using isocentric target doses of either 0, 10, 20, 30, 40, 60, 80, 100, or 120 Gy (D_max_) to the left (unilateral) 10 Gy to 120 Gy (D_max_) using a 7.5 mm collimator. The animals were followed for nine months with MR imaging sequences to assess for structural changes and with FDG-PET as an indicator of metabolic activity, both every three months. In a demonstration of bimodal effects of focally delivered radiation on neural metabolic activity, it was shown that the ratio of PET standardized uptake value at the targeted M1 relative to the non-targeted contralateral side was significantly lowered from baseline at a dose of 60 Gy or greater at nine months post-radiation. By contrast, doses of 10 to 40 Gy (but not 0 Gy) yielded increased PET signal relative to the contralateral. A lesion using T2-weighted MR imaging was visible only in the animal treated with 120 Gy at nine months post-radiation (Figure 1) [34]. No changes in motor function were observed. While exploratory, further study of these animals with histological analysis will provide insight into dose tolerance thresholds with respect to structural tissue changes (pending publication, personal communication).

Clinical studies that suggest a radiomodulation effect

There are also observations in human patients that suggest radiation-based neuromodulation. For example, a subset of patients being treated for trigeminal neuralgia, for unclear reasons, experience near-immediate pain relief following radiosurgery. By contrast, the known cellular changes expected from this procedure require several months to manifest. This observation suggests that radiosurgery may modulate neuronal transmission in grossly intact neurons. Additional clinical evidence may be seen following radiosurgical treatment of brain arteriovenous malformation (AVM) with associated seizures. Post-procedure, seizure frequency decreases, despite the persistence of residual AVM. It may be that the seizure focus in the neuronal tissue around/within the malformation is affected by the radiation dose received, independent of the intended long-term sclerotic effect on the vascular tissue.

Some key clinical observations that suggest a radiomodulation effect in humans are summarized in Table 2.

**Table 2:** A summary of supporting clinical literature supporting a radiomodulation effect. AVM: arteriovenous malformation; MTLE: mesial temporal lobe epilepsy; SRS: stereotactic radiosurgery

Reference	Paradigm	Effect observed
Smith et al. [36]	SRS for trigeminal neuralgia	Immediate effect of SRS on trigeminal nerve pain
Borchers et al. [37]		
Gorgulho et al. [38]		
Steiner and Lindquist [39]	AVM with seizures	SRS for AVMs reduces seizure frequency
Steiner et al. [40]		
Barbaro et al. [41]	Pharmacoresistant unilateral MTLE at 24 Gy to the 50% isodose	SRS reduces seizure frequency
Warnke et al. [42]	Interstitial radiosurgery of seizure-generating gliomas	Radiation increases density of benzodiazepine receptors at the target

There is growing evidence that focal brain activity may be modulated via radiosurgery in the absence of a visible lesion on MR imaging or computed tomography. Radiomodulation studies to date, however, have been designed in narrowly constructed paradigms, and there remains much to be learned. While both the animal and human studies offer clues, they are disparate in their designs and execution. Perhaps most conspicuously, they vary greatly in terms of dose, treatment volume, anatomical target, and intended effect. Limitations in our understanding include knowledge of the lower threshold of the dose required to modulate specific nodes within specific brain circuits and the upper thresholds at which cellular damage and lesion formation occurs. Is the gray matter of the superficial cortex of the same or different radiosensitivity than the cingulate cortex or subcortical nuclei? The long time course required to evaluate for the development of post-radiation changes, particularly at the histological level, also makes investigation challenging and necessary. Other hurdles to adoption include direct comparison to existing approaches, namely, DBS. While focal radiation is not “reversible” like DBS, it does not share the same surgical risks as passing an electrode deep within the brain. With a better understanding of effective doses and targets, focal radiation may prove to be an effective neuromodulation tool.

## Conclusions

Radiomodulation may provide an opportunity for the non-invasive treatment of dysfunctional brain circuits in human disorders. In principle, this would permit the neuromodulation of targeted brain circuits in functional conditions including movement disorders and some psychiatric disorders. Much work remains to be done in order to define necessary parameters and brain targets.
